# Clinical and financial impact of sleep disordered breathing on heart failure admissions

**DOI:** 10.1007/s11325-023-02813-4

**Published:** 2023-03-17

**Authors:** Rami N. Khayat, Kyle Porter, Robin E. Germany, Scott W. McKane, William Healy, Winfried Randerath

**Affiliations:** 1 Division of Pulmonary and Critical Care Medicine, The UCI Comprehensive Sleep Center, University of California-Irvine, 20350 SW Birch Street, Newport Beach, CA 92660 USA; 2 The Ohio State University Sleep Heart Program, Columbus, OH USA; 3 ZOLL Medical, Minnetonka, MN USA; 4 Division of Cardiovascular Diseases, University of Oklahoma, Oklahoma City, OK USA; 5 Division of Pulmonary, Critical Care, and Sleep Medicine, The Medical College of Georgia at Augusta University, Augusta, GA USA; 6 Centre of Sleep Medicine and Respiratory Care, Clinic for Pneumology and Allergology, Bethanien Hospital, Institute of Pneumology at the University of Cologne, Solingen, Germany

**Keywords:** Central sleep apnea, Heart failure, Hospitalization, Obstructive sleep apnea, Readmissions, Sleep disordered breathing

## Abstract

**Background:**

The impact of sleep disordered breathing (SDB) on heart failure (HF) is increasingly recognized. However, limited data exist in support of quantification of the clinical and financial impact of SDB on HF hospitalizations.

**Methods:**

A sleep-heart registry included all patients who underwent inpatient sleep testing during hospitalization for HF at a single cardiac center. Readmission data and actual costs of readmissions were obtained from the institutional honest broker. Patients were classified based on the inpatient sleep study as having no SDB, obstructive sleep apnea (OSA), or central sleep apnea (CSA). Cumulative cardiac readmission rates and costs through 3 and 6 months post-discharge were calculated. Unadjusted and adjusted (age, sex, body mass index, and left ventricular ejection fraction) modeling of cost was performed.

**Results:**

The cohort consisted of 1547 patients, 393 (25%) had no SDB, 438 (28%) had CSA, and 716 (46%) had OSA. Within 6 months of discharge, 195 CSA patients (45%), 264 OSA patients (37%), and 109 no SDB patients (28%) required cardiovascular readmissions. Similarly, 3- and 6-month mortality rates were higher in both SDB groups than those with no SDB. Both unadjusted and adjusted readmission costs were higher in the OSA and CSA groups compared to no SDB group at 3 and 6 months post-discharge with the CSA and OSA group costs nearly double (~ $16,000) the no SDB group (~ $9000) through 6 months.

**Interpretation:**

Previously undiagnosed OSA and CSA are common in patients hospitalized with HF and are associated with increased readmissions rate and mortality.

## Introduction

Heart failure (HF) affected 6.9 million people in the USA in 2020 and is expected to increase by 24% in 2030 based on population growth [1, 2]. Hospital admissions for HF are estimated to cost more than $30 billion in indirect and direct medical costs [2]. A recent review of 87 studies suggested that the median heart-failure specific hospitalization costs $13,418 per patient and the annual medical costs of heart failure care are $24,383 per patient [3]. Costs were observed to be higher for those with a reduced ejection fraction [3]. Despite recent advances in the medical management of HF, almost one quarter of patients hospitalized for acute decompensated HF (ADHF) are readmitted within 1 month of discharge, and more than 50% are readmitted by 6 months [4, 5]. Identification of predictors of readmissions and mortality in HF can help identify high risk patients to receive targeted post-discharge management approaches.

Sleep disordered breathing (SDB), encompassing both central sleep apnea (CSA) and obstructive sleep apnea (OSA), leads to neurohumoral and circulatory perturbations that can potentially decompensate stable HF [6–8]. SDB is associated with myocardial remodeling, sympathetic nerve activation, endothelial dysfunction, oxidative stress, and promotion of arrhythmia [6]. Studies have found that SDB is present in up to 70% of all hospitalized patients with ADHF [9, 10]. Both types of SDB mostly persisted at 6 months post-discharge despite targeted treatment of HF indicating that OSA and CSA may have an independent impact on HF [10–12]. Unsurprisingly, newly diagnosed OSA and CSA were later found to be associated with significant independent effect on post-discharge mortality and readmissions in HF [11, 13].

To date, surveillance for SDB in HF patients is not part of the standard of care, and optimal timing and methods of testing and treatment lack evidence. Current management guidelines for SDB in patients with HF require expensive and repeated testing in the sleep laboratory to be done only during periods of clinical stability [14, 15]. Furthermore, testing is limited to patients who present with symptoms typical for SDB in the general population, despite the widely accepted notion that patients with HF do not have the typical SDB clinical symptom profile [16], thereby leading to under-diagnosis and under-treatment in a population that is most susceptible to the impact of SDB [17]. Testing for SDB during HF hospitalization may provide an opportunity to identify patients at higher risk for readmissions and higher potential for treatment benefit as recent studies have reported [18, 19]. Adapting inpatient surveillance approaches to SDB in hospitalized patients with HF requires better understanding of the clinical and economic burden of SDB and potential cost savings associated with such surveillance. Thus far, however, the financial impact of SDB in HF on the healthcare system, specifically hospitalization related costs, has not been reported previously. In this study, we leveraged our previously established registry of patients with HF who were diagnosed with SDB during an HF hospitalization to provide a comprehensive report of the impact of SDB on mortality, readmissions, and associated costs of readmissions.

## Methods

A registry of patients who were hospitalized with HF at a single academic center and underwent testing for SDB was used to obtain hospitalization costs in patients with and without SDB. The Ohio State University (OSU) Sleep Heart Program maintained an active testing program for SDB between 2007 and 2017. The cohort used in this study included all previously undiagnosed patients who underwent sleep testing during their HF hospitalization between January 2008 and March 2016, the close-out date for this observational analysis.

The inpatient SDB testing program and procedures were discussed previously [10, 20]. Briefly, all patients who were hospitalized with HF at a single cardiovascular hospital received inpatient sleep testing as part of their inpatient HF care. Once the patient deemed stabilized, typically in the first 2–4 days of admission, sleep studies were done in the patients’ room using standard cardiorespiratory devices (Stardust II; Respironics, Murrysville, PA) attended by trained night shift nurses who documented the patient’s sleep time and any interruptions to sleep. Readmissions and their associated costs (actual billing) were tracked for 6 months post-discharge and mortality was followed for a median of 4 years using the OSU Honest Data Broker (OSU Information Warehouse), along with the state vital statistics database as described previously [11].

SDB was defined as an apnea hypopnea index (AHI) ≥ 15 events/h. An AHI cutoff of 15 events/h was selected for the in-hospital study to mitigate against an expected increase in respiratory control instability during the heart failure episode and validated against outpatient polysomnography as previously reported [10, 11, 13]. Respiratory event scoring and classification of predominant OSA and CSA was done according to standard clinical guidelines [21] with OSA diagnosed if more than 50% of the events was obstructive; and CSA if ≥ 50% of the events was central. Heart failure patients with AHI ≤ 15 events/h served as our comparison (no SDB group) to the SDB groups.

Partial data from this cohort were published previously addressing mortality in patients with HFrEF [11] and readmissions related to CSA also in patients with HFrEF [13]. The dataset in the current study includes all the patients in this cohort who have HF regardless of LVEF and address mortality, readmissions, and cost.

### Data collection and outcome measurements

The readmissions and mortality data were generated by the institutional honest broker (“the OSU Information Warehouse”). Only cardiac readmissions were included in the data query using International Classification of Diseases-9 relevant cardiovascular codes. Data coordinators, who were not directly aware of the patient’s SDB status, confirmed the cardiac nature of every admission by reviewing the medical records as previously reported. Elective admissions and admissions for prescheduled procedures were excluded. After the readmissions were verified by the data coordinators, the data set was sent back to the Information Warehouse to obtain the financial data. Admission costs were provided by the hospital financial team and represented actual costs. The study protocol was approved by the OSU Institutional Review Board (2007H0043) and is listed under clinical trials number NCT00701038. This study complies with the Declaration of Helsinki.

### Analysis

Results are presented as mean (standard deviation or interquartile range) for continuous variables and number (%) for categorical variables. Results for readmission costs within 3 months and 6 months were analyzed separately. Cumulative readmission costs within those periods were calculated as the primary outcome. Patients were classified as having no SDB (AHI < 15 events/h), OSA, or CSA. A two-part model was used to model first the probability of a readmission (within 3 or 6 months) and second the estimated cumulative readmission cost, conditional on having at least one readmission. The full two-part model calculates an estimated cost per patient utilizing results from both parts. In our application, the first part was a probit regression model, and the second part was a generalized linear model with a log link and gamma distribution. An unadjusted univariable model was fit followed by a multivariable model adjusting for characteristics with high absolute standard difference between groups.

Multiple imputation was employed to account for missing cost values (log-transformed) and also missing values of covariates in the multivariable two-part model. Rubin’s rules were used to combine imputation analysis results in order to obtain correct confidence intervals and *p* values.

## Results

### Participants’ characteristics

Of the 1547 patients included in the analysis, 393 (25%) had no SDB, 438 (28%) had CSA, and 716 (46%) had OSA. Characteristics for the groups are displayed in Table 1 and comparisons are reported using absolute standardized differences. The prevalence and type of SDB in this population were similar to our previous findings [10] and other reports in similar populations [22]. The only differences across the comparisons were in age, sex, and left ventricular ejection fraction, which are risk factors for SDB in this population [9, 10].

**Table 1:** Baseline characteristics

Characteristic	No SDB (*n* = 393)	CSA (*n* = 438)	OSA (*n* = 716)	Absolute standardized differences		
				No SDB vs. CSA	No SDB vs. OSA	CSA vs. OSA
Age, mean (SD)^a^	55.3 (15.0)	60.1 (14.6) {1}	60.7 (13.3) {1}	0.32	0.39	0.05
Sex, male	203 (13%)	356 (23%)	492 (32%)	0.66	0.35	0.29
BMI, mean (SD)	30.3 (7.9) {20}	29.7 (7.5) {21}	29.7 (7.5) {21}	0.08	0.29	0.37
AHI, mean (SD)	9.3 (4.3)	47.4 (17.5)	36.1 (16.3)	2.99	2.26	0.67
LVEF, mean (SD)	38.7 (16.0) {13}	28.6 (15.7) {24}	35.0 (17.1) {8}	0.63	0.22	0.39
Creatinine, median (IQR)	0.98 (0.80–1.25) {2}	1.20 (0.97–1.63) {2}	1.16 (0.89–1.57) {1}	0.34	0.26	0.06
BUN, median (IQR)	16 (12–24) {2}	21 (14–31) {3}	20 (14–31) {2}	0.30	0.28	0.02
Atrial fibrillation	149 (10%) {3}	194 (13%) {7}	354 (23%) {3}	0.14	0.23	0.09
Hypertension	196 (13%) {2}	209 (14%) {7}	410 (27%) {1}	0.03	0.15	0.18
Coronary artery disease	213 (14%) {2}	281 (18%) {7}	392 (32%) {1}	0.22	0.30	0.08
Chronic kidney disease	78 (5%) {3}	155 (10%) {7}	242 (16%) {1}	0.36	0.32	0.04
Diabetes	128 (8%) {2}	162 (11%) {7}	344 (22%) {1}	0.10	0.32	0.21

Standardized differences are reported rather than *p* values to describe differences between groups to use a measure that is independent of sample size and to minimize use of *p* values. Standardized differences of 0.2 or smaller are considered small and 0.2 to 0.5 are moderate. Most of the standardized differences above are less than 0.4

*AHI* apnea hypopnea index, *BMI* body mass index, *CSA* central sleep apnea, *IQR* interquartile range, *LVEF* left ventricular ejection fraction, *OSA* obstructive sleep apnea, *SD* standard deviation, *SDB* sleep disordered breathing

^a^Number missing in brackets {}

### Effect of SDB on cardiovascular readmissions

**Fig. 1:**
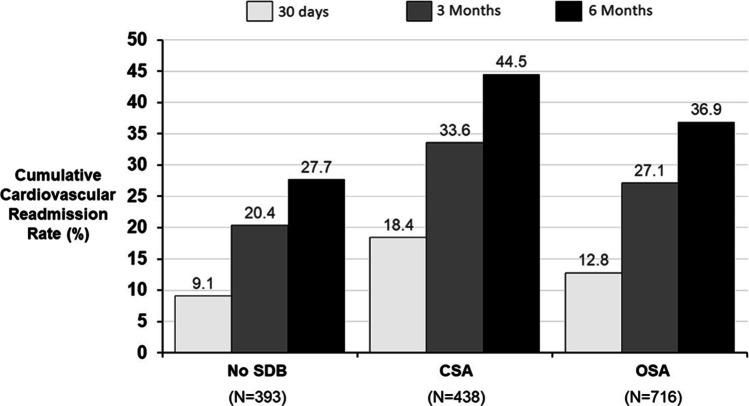
Readmission rates for the three SDB groups. Cardiovascular readmissions are adjusted for age, sex, weight, and left ejection fraction. Abbreviations: CSA central sleep apnea, OSA obstructive sleep apnea, P *p* value, SDB sleep disordered breathing

Both OSA and CSA were independently associated with cardiovascular readmissions at 3 and 6 months after adjustment for sex, age, BMI, and LVEF. Patients with CSA also had more significant increase in readmissions at 1 month compared to those with no SDB (Table 2). Overall, within 6 months of discharge, 195 patients with CSA (45%), 264 patients with OSA (37%), and 109 patients with no SDB (28%) experienced a readmission for a cardiovascular diagnosis (Fig. 1) Adjustment ﻿for sex, age, BMI, and LVEF is reported in Table 2. Other models that were also explored included discharge medications, presence of coronary disease, hypertension, diabetes, and chronic kidney disease. Similar to our previous findings, the effect of SDB on readmissions remained independent at both time intervals [13].

**Table 2:** Adjusted readmission rate through 6 months by SDB status

Readmission rate*	No SDB (*n* = 393)	CSA (*n* = 438)	OSA (*n* = 716)	*p* value OSA vs. no SDB	*p* value CSA vs. no SDB
30 days	33 (9%)	74 (18%)	86 (13%)	0.07	< 0.001
3 months	80 (20%)	147 (34%)	194 (27%)	0.01	< 0.001
6 months	109 (28%)	195 (45%)	264 (37%)	0.002	< 0.001

*CSA* central sleep apnea, *OSA* obstructive sleep apnea, *SDB* sleep disordered breathing

^*^At least 1 readmission within stated range (30 day, 3 months, or 6 months) of initial discharge

### Effect of SDB on post-discharge mortality

Both CSA and OSA were associated with increased mortality in the 6 months after discharge (Table 3). Patients with CSA had significantly higher mortality rate than those with no SDB at 1, 3, and 6 months. Patients with OSA had higher mortality rate than those with no SDB at 3 and 6 months. The association between both OSA and CSA and post-discharge mortality was present after adjusting to the characteristics reported in Table 1.

**Table 3:** Adjusted mortality through 6 months by SDB status

Mortality rate	No SDB (*n* = 393)	CSA (*n* = 438)	OSA (*n* = 716)	*p* value OSA vs. no SDB	*p* value CSA vs. no SDB
30 days	10 (3%)	23 (6%)	32 (5%)	0.138	0.0051
3 months	18 (5%)	45 (10%)	56 (8%)	0.044	0.002
6 months	26 (7%)	64 (15%)	88 (12%)	0.003	< 0.001

*CSA* central sleep apnea, *OSA* obstructive sleep apnea, *SDB* sleep disordered breathing

### Impact of SDB on readmission cost

The institutional Information Warehouse search provided 633 readmissions in 421 unique patients through 3 months and 1060 readmissions from 568 unique patients within 6 months. Hospital billing data for the index admission and all readmissions at 6 months were available for 892 (84%) of all the readmissions. The remaining readmissions with missing costs (16%) were imputed.

**Fig. 2:**
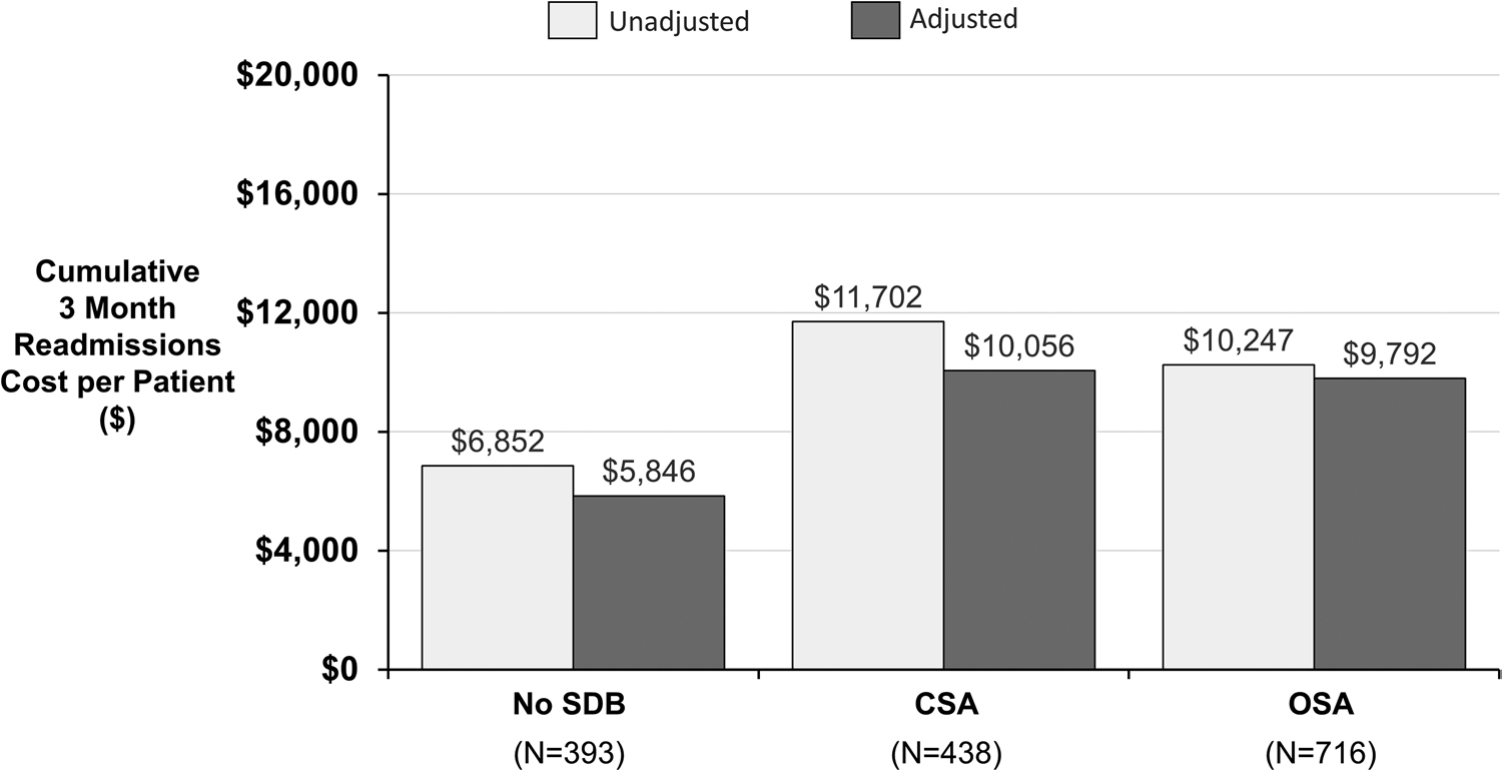
Estimated cumulative 3-month readmission cost per patient. Estimated cost and 95% confidence intervals are provided for the unadjusted model and model adjusted for age, sex, body mass index, and left ventricular ejection fraction. Abbreviations: CSA central sleep apnea, OSA obstructive sleep apnea, P *p* value, SDB sleep disordered breathing

**Fig. 3:**
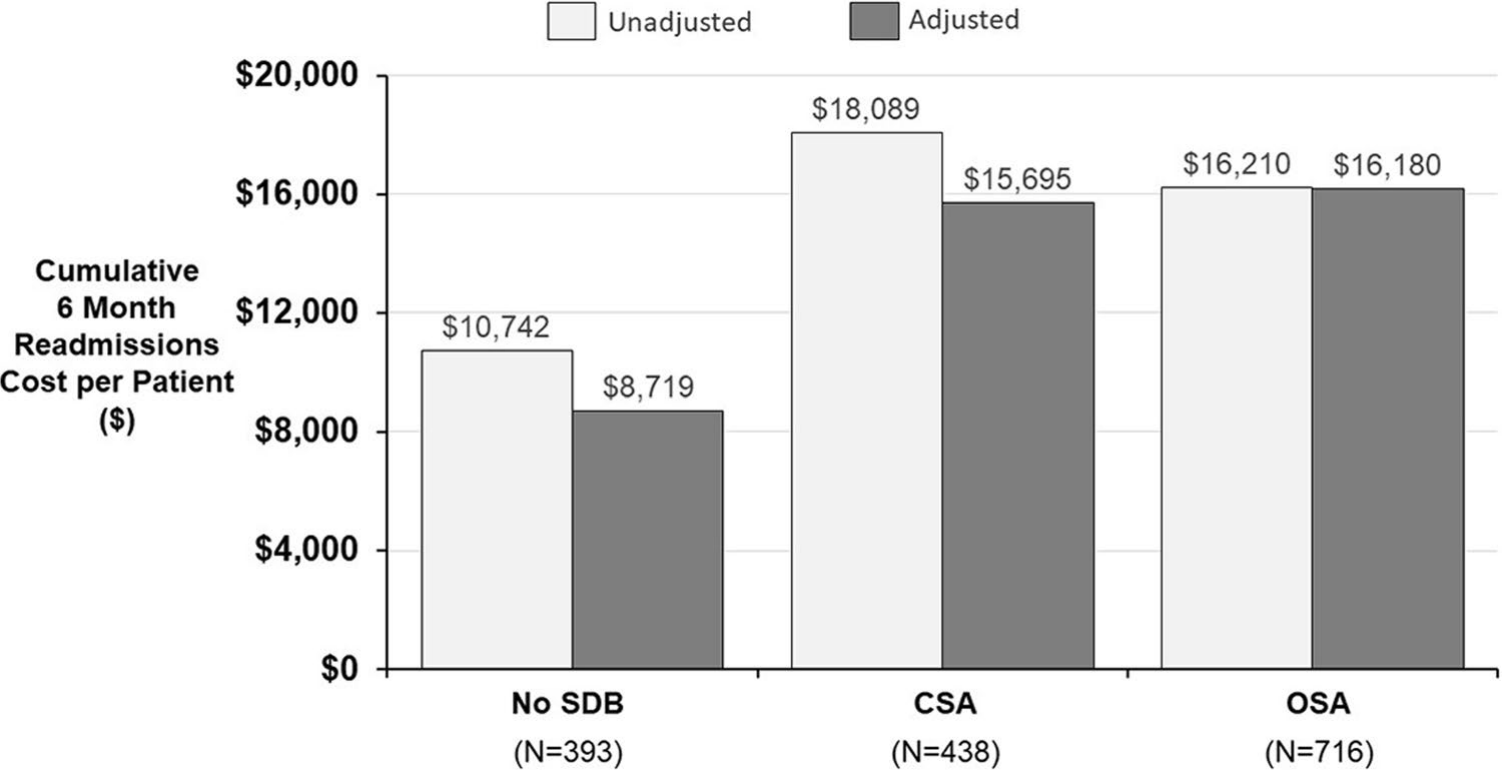
Estimated cumulative 6-month readmission cost per patient. Estimated cost and 95% confidence intervals are provided for the unadjusted model and model adjusted for age, sex, body mass index, and left ventricular ejection fraction. Abbreviations: CSA central sleep apnea, OSA obstructive sleep apnea, P p value, SDB sleep disordered breathing

The cumulative readmission cost was increased in patients with either OSA or CSA compared to no SDB through 3 months (Fig. 2) and 6 months (Fig. 3). Unadjusted costs were highest in patients with CSA at both 3 and 6 months ($11,702 and $18,089, respectively), followed by OSA ($10,247 and $16,210) and no SDB ($6852 and $10,742). When adjusted for age, sex, body mass index, and left ventricular ejection fraction, the difference between OSA and CSA was < $1000 at 3 and 6 months (each approximately $10,000 at 3 months and $16,000 at 6 months); however, both remained almost double the no SDB group after adjustment ($5846 at 3 months and $8719 at 6 months). Regardless of type of SDB, there was a meaningful increase in hospital charges in patients with SDB at 3 and 6 months (Fig. 3).

The effect of SDB treatment on the cost of readmissions was also considered. There were 327 patients who were confirmed to be on treatment in the first-year post-discharge. The average time to initiation of treatment was 3.7 ± 4.3 months, meaning more than half of those patients likely did not start treatment prior to the 3-month time point although the majority did prior to the 6-month endpoint, and compliance information was not available. Therefore, we elected to keep these patients in the analysis to minimize potential bias related to access and acceptance of positive airway pressure therapy in some patients.

## Discussion

In this large prospective cohort reporting the clinical and economic impact of SDB on HF hospitalizations, we found an independent impact of both OSA and CSA on mortality and readmissions. Furthermore, the study demonstrates significantly increased cost of HF readmissions attributable to having both OSA and CSA compared to having no SDB. The increased cost associated with having OSA or CSA was present at both 3 and 6 months. This financial impact of OSA and CSA had an adjusted 6-month cost near $16,000 compared to less than $9000 for those without SDB.

In this study, we also reported the independent relationship between newly diagnosed SDB during HF admission and post-discharge readmissions and mortality. We note that a portion of these patients was included in a previous publication addressing mortality only [11]. The current findings are consistent with our previously published studies in the same cohort. Nevertheless, these findings are confirmatory to our previous studies as well as others and support the impact of SDB on clinically important post-discharge outcomes [11, 13, 19, 23].

The finding of increased cost of hospitalizations associated with having CSA or OSA in this population could be expected given the impact of SDB on readmissions demonstrated previously [13] and again in this current study. However, an accurate estimate of the financial impact of these readmissions is critical for modeling the SDB disease-impact and the cost of interventions to minimize readmissions. It must be noted here that hospitalization cost is only one aspect of the overall economic cost of disease. Other important components that are not addressed in the study include loss of workdays, impact on productivity and early retirement, and unscheduled outpatient and emergency visits, to list a few.

While OSA and CSA are common and associated with high mortality in HF patients, SDB is often not identified in this population. In one retrospective study of Medicare files, only 2% of newly diagnosed HF patients were tested for SDB [24]. The low level of screening may be partially attributable to the fact that the symptoms of sleep apnea often mirror those of heart failure including fatigue, sleepiness, and depression [16]. Thus, sleep testing is often missed based on lack of excessive daytime sleepiness [16, 17]. This study did not address the effect treatment of SDB in the post-discharge phase on reducing the cost of readmissions. However, a recent trial evaluating inpatient initiation of positive airway pressure therapy for hospitalized HF patients with newly diagnosed OSA demonstrated a decrease in 6-month readmissions [19]. Other, albeit observational, studies demonstrated improved mortality or readmissions in the post-discharge phase in patients with OSA and CSA who were treated post-discharge [11, 25]. More robust evidence of treatment benefit from randomized controlled trials remains lacking, however. Although inpatient sleep testing is not currently reimbursed, surveillance programs can easily be established using low-cost testing approaches similar to the one used in this study [26].

### Limitations

The limitations of this study include the use of cardiorespiratory testing in the hospitalized setting. Our inpatient testing method has been previously validated against polysomnography [27] and is accepted for the diagnosis of OSA in this population [28]. We have validated the sensitivity for CSA of this method in hospitalized heart failure patients as well [10] with similar approaches widely used in this population [9].

We have increased the precision of inpatient sleep testing by using visual inspection of sleep, higher AHI threshold, and a validated interpretation approach [10–12]. The billing dataset was derived from a single center institution billing procedures with various degrees of generalizability to different institutions. The study intentionally did not adjust for the impact of treatment of SDB in the post-discharge settings. Whether or not successful treatment of SDB in this setting is associated with a positive impact on HF hospitalization remains unknown. Therefore, keeping potentially treated patients in the analysis could only make our estimates conservative. All patients with SDB were indeed recommended to follow up with the sleep clinic at our institution or with their local physician for additional testing and treatment. We have previously estimated that the time to conduct repeated testing and establishing effective treatment was about 3 months [11], which would have minimal impact on our 6-month follow-up financial data. Also, only including hospitalizations from within one hospital system could lead to an underestimate of financial costs related to the HF admissions for all the groups, again making the financial estimates potentially more conservative.

## Conclusion

This study provides a framework to the understanding of the mortality, HF readmission, and added financial impact of SDB on HF patients. This study sheds light on the full impact of SDB on HF by including clinical and financial outcomes. The findings of this study can be used to model the financial impact of interventions that target the impact of SDB on heart failure. Of course, studies that address the clinical impact of SDB treatment on HF hospitalizations will further address whether or not treatment of SDB will decrease the SDB attributed cost of HF admissions.

## Data Availability

The datasets generated during and/or analyzed during the current study are available from the corresponding author on reasonable request.

## Abbreviations

AHI: Apnea hypopnea index
CSA: Central sleep apnea
HF: Heart failure
OSA: Obstructive sleep apnea
SDB: Sleep disordered breathing
